# Sex-specific effects of peptidyl arginine deiminase 4 deficiency in the cafeteria diet-induced obesity-associated metabolic complications

**DOI:** 10.3389/fendo.2025.1694559

**Published:** 2026-01-02

**Authors:** Andrej Feješ, Emil Bečka, Ivana Feješ Slivková, Kristína Macáková, Emőke Šteňová, Michal Pastorek, Peter Celec, Roman Gardlík, Katarína Šebeková, Veronika Borbélyová

**Affiliations:** 1 Institute of Molecular Biomedicine, Faculty of Medicine, Comenius University, Bratislava, Slovakia; 2 1^st^ Department of Internal Medicine, Faculty of Medicine, University Hospital, Comenius University, Bratislava, Slovakia

**Keywords:** gender, insulin resistance, metabolic syndrome, neutrophil extracellular traps, thermogenesis, steatohepatitis

## Abstract

**Background:**

Obesity is a global health challenge linked to chronic non-communicable diseases. Low-grade inflammation and altered immune responses, including neutrophil extracellular trap (NET) formation, contribute to metabolic complications. Peptidyl arginine deiminase 4 (PAD4) is critical for NET formation, and its inhibition shows therapeutic potential. However, the sex-specific effects of PAD4 deficiency in diet-induced obesity remain unexplored.

**Methods:**

This study investigated the impact of PAD4 deficiency on obesity-related metabolic pathologies in male and female C57BL/6 wild-type (WT) and Pad4^(-/-)^ mice (n=5-6/group) fed a 22-week obesogenic cafeteria (CAF) diet. We hypothesized that PAD4 deficiency would ameliorate obesity-related metabolic and behavioral complications for both sexes. Body weight and composition, glucose and lipid metabolism, liver damage markers, and behavior were assessed. NETs were quantified via flow cytometry.

**Results:**

Pad4^(-/-)^ males on CAF diet exhibited delayed obesity onset, lower body weight gain, and improved dyslipidemia than WT CAF males. This was associated with enhanced metabolic adaptation, indicated by higher brown adipose tissue temperature in Pad4^(-/-)^ males. Conversely, Pad4^(-/-)^ females on the CAF diet showed comparable weight gain to WT CAF females, similar or worsened dyslipidemia, impaired glucose metabolism, and higher liver lipid accumulation. While WT CAF females showed increased brown adipose tissue temperature, Pad4^(-/-)^ CAF females did not.

**Conclusion:**

PAD4 deficiency exerts sex-specific effects on obesity-related metabolic complications in mice. Inhibition of NET formation appears protective in males but not females on an obesogenic diet. These findings underscore the importance of considering sex as a biological variable in obesity research and developing sex-specific therapies.

## 1 Introduction

Obesity is a chronic, multifactorial disease defined by extensive fat accumulation that increases the risk of developing chronic non-communicable diseases (1). The prevalence of overweight and obesity represents about 30% of the world’s population, with a rising tendency of mortality from cardiovascular events, with obesity as a primary contributor (2, 3). Visceral adiposity is associated with low-grade inflammation that increases the manifestation of metabolic complications (4). Hypertrophic adipocytes secrete adipokines, which, in turn, promote the production of pro-inflammatory cytokines that contribute to oxidative stress, macrophage polarization, and insulin resistance (5–8). These pathologies may induce hypoxia, and adipocytes undergo apoptosis and necrosis, with their cellular structures being expelled into the extracellular space, where they act as damage-associated molecular patterns (DAMPs). This process induces neutrophil tissue-specific migration, neutrophil DAMPs recognition, and the formation of neutrophil extracellular traps (NETs) (9), thereby enhancing low-grade inflammation. During NET formation, neutrophils release DNA into the extracellular space (ecDNA) along with neutrophil-specific proteins, mainly neutrophil myeloperoxidase (MPO) and neutrophil elastase (9). Peptidyl-arginine deiminase 4 (PAD4) is described as one of the key enzymes involved in NETosis (10), a process that includes histone citrullination, promoting intracellular chromatin decondensation, thereby facilitating the expulsion of intracellular DNA coated with antimicrobial molecules into the extracellular space (11).

Previous studies have shown higher NET-associated markers in patients with obesity (12, 13) however, their role remains unclear. In preclinical *in vivo* research, the role of PAD4 in NETs formation is studied through pharmacological blocking (e.g., Cl-amidine) or the use of knockout animals. This approach alleviates signs of rheumatoid arthritis (14), sepsis (15), thrombosis (16), type 1 diabetes (17), and obesity (18). Additionally, MPO- and neutrophil elastase-knockout mice are used to evaluate the role of NETs in metabolic disorders. MPO deficiency improved body weight gain and insulin sensitivity (19) and suppressed the formation of atherosclerotic plaques (20) in mice fed a high-fat diet (HFD). Furthermore, neutrophil elastase deficiency enhanced body weight gain, vascular permeability, leukocyte extravasation (21), and vascular leakage (22) in HFD mice. Most of the above studies were conducted in male mice. A recent study demonstrated improved body weight gain in HFD-fed Pad4^(-/-)^ females and males however, data on reduced insulin resistance and cardiac remodelling were only shown for males (18).

Due to the different pathophysiology of obesity-related cardiometabolic complications between women and men (23, 24), the National Institute of Health and the European Medicines Agency recommend using both sexes of rodents in preclinical research (25). Since females are generally understudied, this study aimed to investigate potential sex differences in obesity-related metabolic pathologies in Pad4^(-/-)^ mice subjected to a chronic obesogenic cafeteria (CAF) diet challenge. We hypothesised that Pad4 deficiency would ameliorate obesity-related metabolic and behavioural complications in both sexes, as evidenced by studies using HFD, which showed no sex differences in body weight gain.

## 2 Materials and methods

### 2.1 Animals

The Pad4-deficient females and males (n=11/11) were obtained from a breeding pair of homozygous Pad4 knockout mice (B6. Cg-Padi4tm1.1Kmow/J; Jackson Laboratory). Age-matched C57BL/6J females and males (n=11/12; Jackson Laboratory) at six months of age were used as wild-type controls. Animals were group-housed (2 per cage) in an open cage system (20x12x20 cm), kept in a controlled environment (temperature: 24 ± 2 °C, humidity: 55 ± 10%, 12-hour light/dark cycle), with ad libitum access to food and water.

#### 2.1.1 Experimental design, diet composition, and body weight measurement

**Figure 1:**
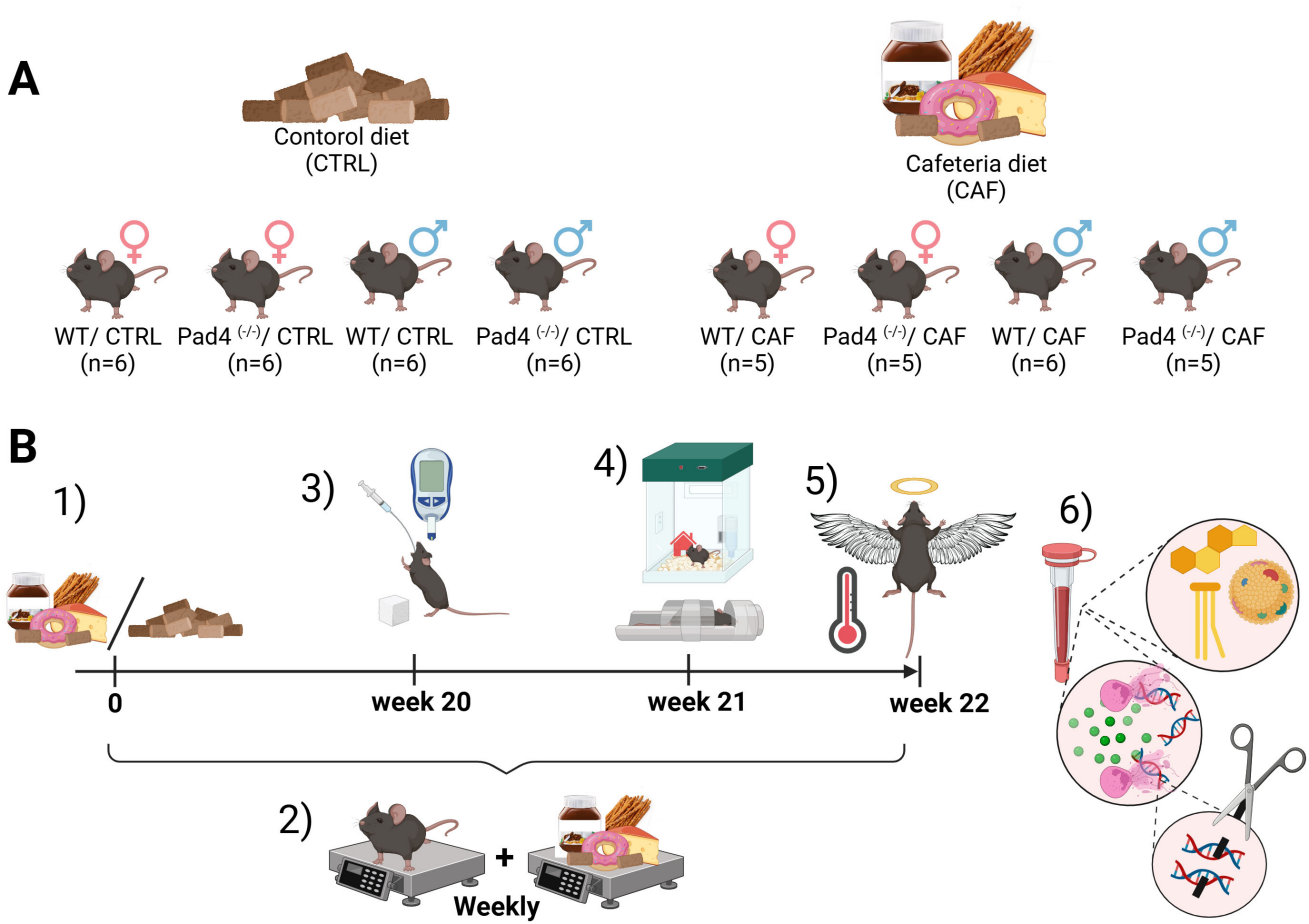
**(A)** Experimental groups; **(B)** Design of the experiment: 1) Administration of cafeteria/control diet; 2) Measurements of body weight (weekly) and caloric intake (in 2-day intervals); 3) Oral glucose tolerance test performed at week 20; 4) Behavioral testing and densitometric measurements conducted at week 21; 5) Body temperature measurements 2-days before sacrifice. On week 22, animals were euthanized, and blood and organs were collected. 6) Analytical phase of the experiment. Created with Biorender.com.

Mice of both sexes were randomized according to body weight and assigned into the following groups: CTRL animals of WT and Pad4^(-/-)^ mice were fed a standard diet (Ssniff V1534–000 R/M-H- maintenance diet, Ssniff Spezialdiäten GmbH, Germany). For the CAF diet, two menus were created (**Supplementary Table 1**), each containing one salty, one processed, and three sweet food items with different textures (e.g., soft, crunchy, smooth, rigid, or chewy), as well as a standard diet (26). Menus were changed every other day. The total caloric intake during the 22-week experiment was calculated based on the nutritional values provided by the manufacturer. Body weight was monitored weekly. Body weight gain was calculated as the difference between the initial and terminal body weight (%). Food efficiency was calculated as the ratio between body weight gain and net energy intake. The animals underwent oral glucose tolerance tests (oGTT), “home-cage” monitoring of behavior in week 20, body composition analysis in week 21, and thermal imaging 2 days before the end of the experiment. Experimenters conducting behavioral, metabolic, and physiological assessments were blinded to genotype and diet during data collection. The experiment was terminated in week 22 (**Figure 1B**). To minimize handling-induced stress, estrous cycle staging was not performed in females, which could itself alter behavioral, metabolic, and physiological parameters.

#### 2.1.2 Oral glucose tolerance test

After an 8-hour-long food-deprivation period during the dark phase, fasting plasma glucose (FPG) was measured using a glucometer (Accu-Chek Performa, Roche Slovakia, s.r.o, Diabetes Care, Bratislava, Slovakia), and blood for fasting plasma insulin (FPI; Mouse Insulin ELISA, Mercodia, Uppsala, Sweden) was taken from the tail vein. The insulin sensitivity check index (QUICKI, a species-independent marker of insulin resistance) (27) was calculated: *QUICKI = 1/[log(FPI) + log(FPG)]*; FPI (µIU/mL), FPG (mg/dL) (28). For oGTT, animals were orally gavaged with glucose solution (2 g/kg; D-(+)-Glucose H_2_O p.a., Centralchem, s.r.o, Bratislava, Slovakia). Glycemia was measured 15, 30, 60, 90, and 120 minutes after glucose load (29). The area under the glycemic curves (AUC) was calculated.

#### 2.1.3 Body composition analysis

The fat and lean tissue content was determined using dual-energy X-ray absorptiometry with the Horizon DXA System (Hologic, Marlborough, MA, USA), equipped with software specifically designed for small animals (Hologic APEX Software version 5.6.0.5). The device was calibrated using the Small Animal Step Phantom. Animals were anesthetized using i.p. injection of ketamine (100mg/kg, Richter Pharma AG, Austria) and xylazine (10mg/kg, Ecuphar N.V., Belgium), and bone mineral density (BMD), bone mineral content (BMC), lean and fat mass and abdominal fat mass (analyzed manually) after delineation of ROI between L3-S3 vertebrae were measured (30). The mean of three consecutive measurements was calculated per animal.

#### 2.1.4 Body temperature measurement

Infrared thermography was performed to detect body temperature (Teledyne FLIR-E64501, Wilsonville, OR, USA). During the ocular surface temperature measurements, the mice were immobilized by an experimenter, and three thermal images were recorded per mouse. The distance between the mouse and the camera was 20 cm. Besides ocular surface temperature, the temperature of interscapular brown adipose tissue under inhalation anesthesia (3% isoflurane, Isoflurane 1000 mg/g, Vetpharma Animal Health, Barcelona, Spain, 97% oxygen) was also assessed (two thermal pictures per mouse). The average temperatures from the photographs were used for statistical evaluation (31).

#### 2.1.5 Continuous monitoring of behavior in the PhenoTyper home-cage monitoring system

Animals were individually placed into PhenoTyper cages (45 x 45 cm; Noldus Information Technology, Wageningen, The Netherlands) with bedding material and free access to water, food, and shelter for 24 hours before testing, under a 12-hour light/dark condition. After habituation (lasting 24 hours), a 24-hour-long recording of behavior was conducted. All recordings were automatically analyzed in 30-minute intervals using EthoVision XT 10.0 (Noldus Information Technology, Wageningen, Netherlands). Locomotor activity (m), time spent in the food zone (min), water zone (min), and shelter zone (sleeping and immobility in the shelter zone, min) were analyzed. Total food and water intake was weighted manually. Results are presented separately as the dynamics of observed parameters and as the area under the curve (AUC) calculated for both the light and dark cycles.

#### 2.1.6 Sacrifice and sample collection

At the end of the experiment (week 22), mice were anesthetized via inhalation anesthesia (3% isoflurane, 97% oxygen). Terminal blood was collected from the orbital sinus into EDTA and heparin tubes (Microvette, Sarstedt, Nümbrecht, Germany). Following terminal blood collection, animals were euthanized by cervical dislocation. Blood was centrifuged (1600 g at 4°C for 10 minutes), and plasma was collected and stored at -20°C. The liver was removed, and two 100 mg aliquots were stored at -80°C to determine the total cholesterol and triacylglycerol content.

#### 2.1.7 Determination of neutrophil extracellular trap formation

**Figure 2:**
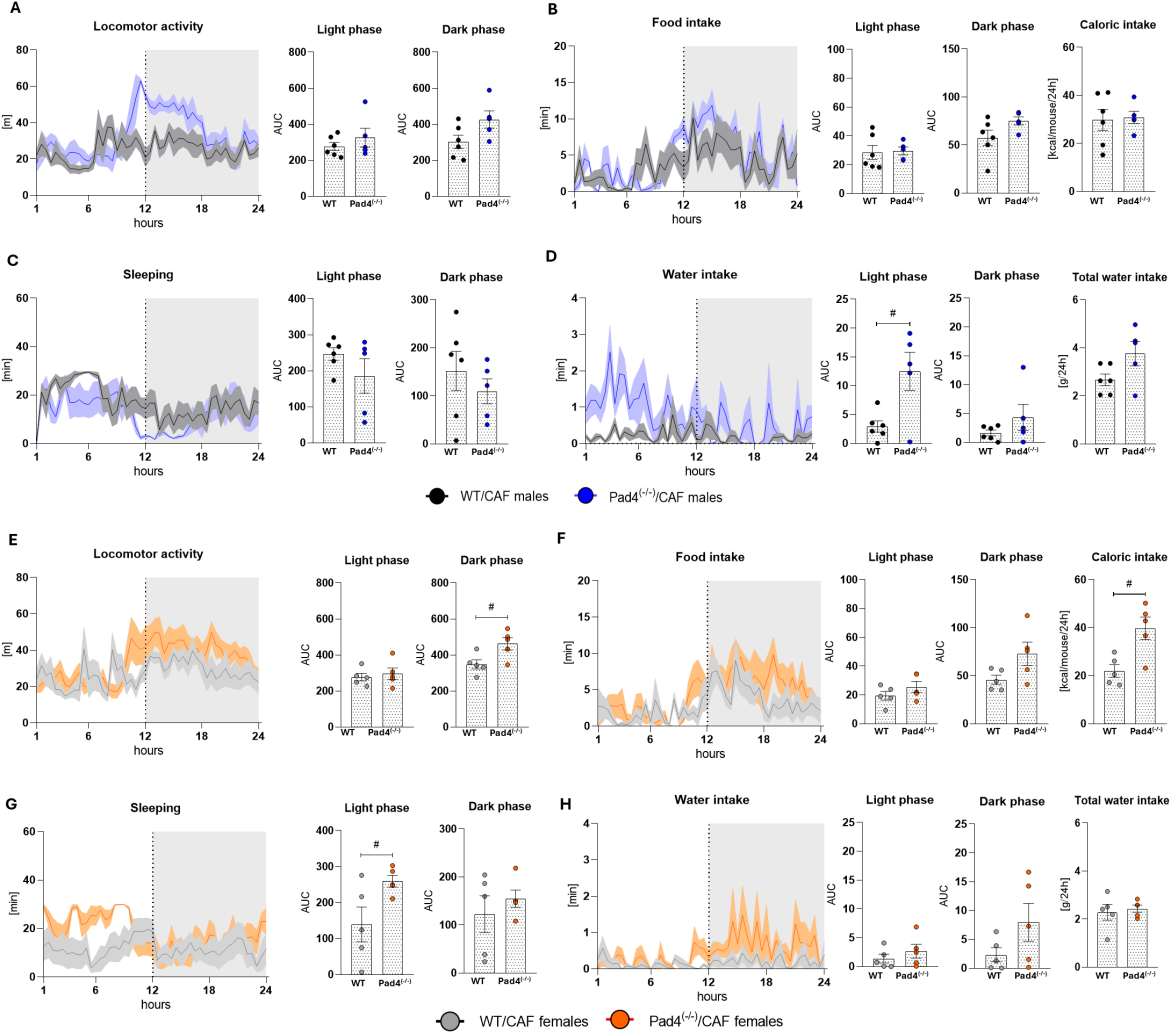
Home-cage monitoring. **(A)** 24-hour locomotor activity and area under the curve (AUC) of locomotor activity during the light (passive) and dark (active) phases of the day cycle in the CAF diet-fed males; **(B)** 24-hours food intake, AUC of food intake during the light and dark phases of the day cycle, and 24-hour caloric intake in the CAF diet-fed males **(C)** 24-hours sleeping behavior, and AUC of sleeping during light and dark phase of the day cycle in the CAF diet-fed males **(D)** 24-hours water intake, AUC of water intake during light and dark phase of the day cycle, and 24-hour total water intake in the CAF diet-fed males; **(E)** 24-hours locomotor activity and AUC of locomotor activity during light (passive) and dark (active) phase of the day cycle in the CAF diet-fed females; **(F)** 24-hours food intake, AUC of food intake during light and dark phase of the day cycle, and 24-hour caloric intake in the CAF diet-fed males **(G)** 24-hours sleeping behavior, and AUC of sleeping during light and dark phases of the day cycle in the CAF diet-fed males **(H)** 24-hour water intake, AUC of water intake during light and dark phases of the day cycle, and 24-hour total water intake in the CAF diet-fed males; WT/CAF males: n=6; Pad4^(-/-)^/CAF males: n=5; WT/CAF females: n=5; Pad4^(-/-)^/CAF females: n=5. Statistical analysis: Calculation of the area under the curve, with the following independent T-test. Data are presented as mean ± SEM. **^#^**WT vs. Pad4^(-/-)^. **^#^**p<0.05.

**Figure 3:**
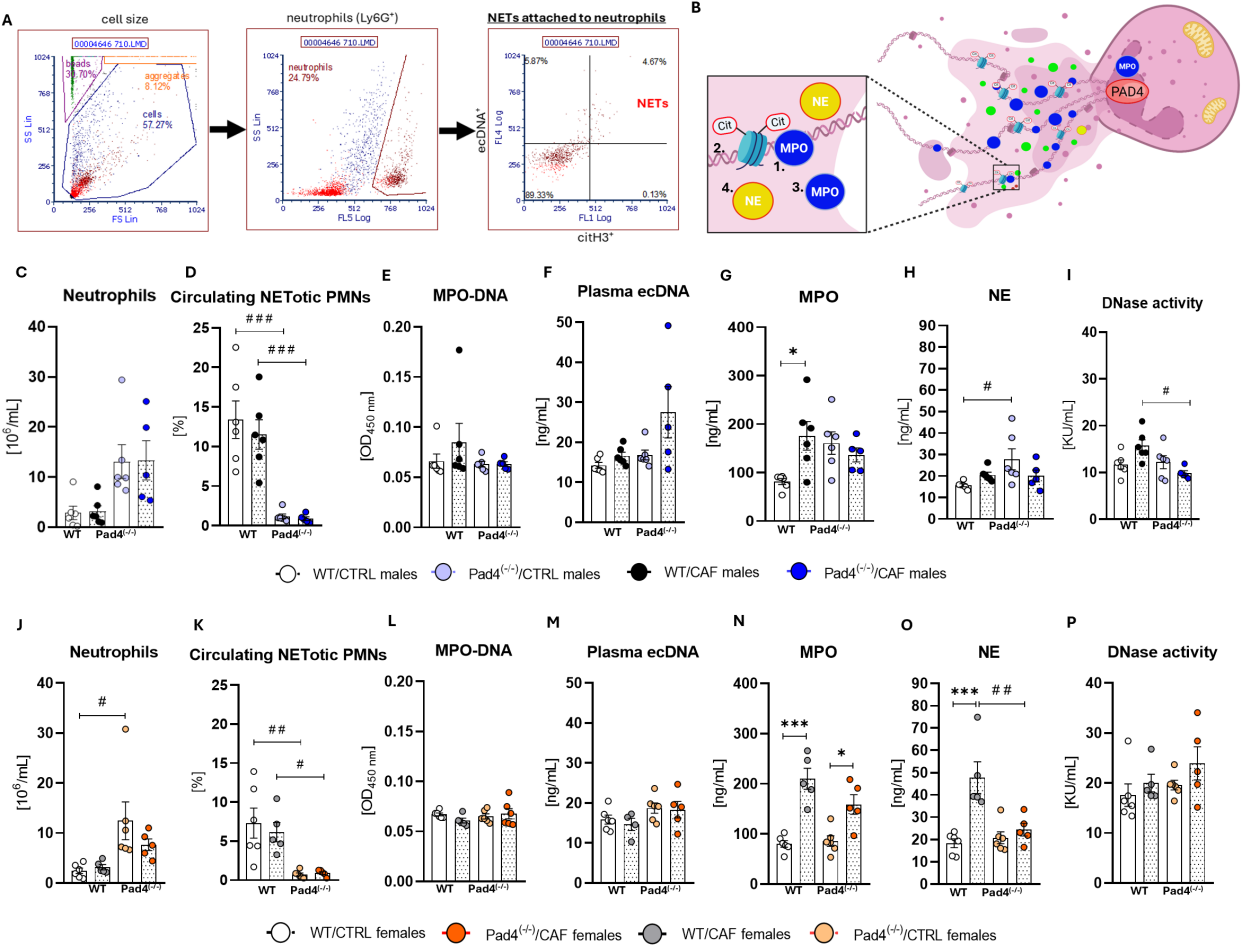
Circulating neutrophil extracellular trap (NET)-related markers. **(A)** Gating strategy for the NETotic PMNs detection; **(B)** Scheme of NETotic PMNs and their main structures 1- Myeloperoxidase-DNA complex; 2- Citrullinated histone; 3- Myeloperoxidase; 4- Neutrophil elastase; **(C)** Neutrophil count in males; **(D)** Circulating NETs in males; **(E)** Myeloperoxidase (MPO)-DNA complex in plasma in males, **(F)** Extracellular DNA (ecDNA) concentrations in plasma of males; **(G)** Plasma concentrations of neutrophil myeloperoxidase (MPO) in males; **(H)** Plasma concentrations of neutrophil elastase (NE) in males; **(I)** Plasma DNase activity in males; **(J)** Neutrophil count in females; **(K)** Percentage of NETs in in females; **(L)** MPO-DNA complex in plasma in females; **(M)** ecDNA concentrations in plasma of females; **(N)** Plasma MPO concentrations in females; **(O)** Plasma NE concentrations in females; **(P)** Plasma DNase activity in females. WT/CTRL males: n=6; WT/CAF males: n=6; Pad4^(-/-)^/CTRL males: n=6; Pad4^(-/-)^/CAF males: n=5; WT/CTRL females: n=6; WT/CAF females: n=5; Pad4^(-/-)^/CTRL females: n=6; Pad4^(-/-)^/CAF females: n=5. Statistical analysis: 2-way ANOVA with the Bonferroni *post-hoc* test. Data are presented as mean ± SEM. ^*^CAF vs. CTRL (for both genotypes); **^#^**WT vs. Pad4^(-/-)^ (for both diet groups). ^*/#^p<0.05; ^**/##^p<0.01; ^***/###^p<0.001. Created with Biorender.com.

50 μl of heparinized blood underwent erythrocyte lysis with ammonium chloride (150 mM NH_4_Cl, 10 mM KHCO_3_, 0.1 mM EDTA) for 15 min, then was centrifuged (400g, 4 °C, 10 min) to obtain the leukocyte fraction. The pellet was resuspended in a staining mixture of 500 nM TO-PRO-3^®^ (ThermoFisher), 0.5 μg/ml PE-Ly-6G (Biolegend), and 0.5 μg/ml anti-Histone H3 (citrulline R2 R8 R17) antibodies (Abcam) in 100 μl RPMI1640 (Biosera) with 1% bovine serum (Biosere). After 15 min incubation at room temperature in the dark, 0.2 μg/ml Alexa Fluor^®^ 488 Donkey anti-rabbit IgG (Biolegend) was added, followed by 15 min incubation and 20 µl CountBrightTM beads (Thermo Fisher). Samples were analyzed using the Cytomics FC500 flow cytometer (Beckman Coulter), collecting data on the FITC (489/515 nm), PE (566/574 nm), and APC (651/660 nm) channels with FCS Express 6.0 (*De Novo* Software). Gating strategy selected Ly6G neutrophil expression without considering the heterogeneous scattering properties of NETs (**Figure 2A**). Total neutrophils were counted using ball counting, and TO-PRO-3^®^/citrullinated histone H3 double-positive cells were identified as NETs, expressed as a percentage of total Ly6G-positive cells (NETotic PMNs, **Figure 3B**).

#### 2.1.8 Isolation and measuring the concentration of circulating ecDNA and DNase activity

Heparinized plasma (100 µl, 1600 g, 10 min, 4 °C) was centrifuged (16,000 g, 10 min, 4 °C), and the supernatant was collected for ecDNA isolation using the QIAcube (Qiagen) with the QIAamp DNA Blood Mini Kit. The ecDNA concentration was measured using the Qubit dsDNA HS assay (Thermo Fisher). The single radial enzyme diffusion method was used to determine DNase 1 activity (KU/mL) (32).

#### 2.1.9 Biochemical analysis

Standard laboratory methods were used to assess plasma triacylglycerols (TAG), total cholesterol (CHOL), high-density lipoprotein-cholesterol (HDL-C), low-density lipoprotein-cholesterol (LDL) concentrations, and aspartate aminotransferase (AST) and alanine aminotransferase (ALT) activity (Biolis 24i Premium analyser, Tokyo Boeki Machinery, Tokyo, Japan). The concentrations of inflammatory cytokines (IL-1α, IL-1β, IL-6, IL-10, IL-17A, IL-23, IL-27, IL-12p70, TNF-α, GM-CSF, MCP-1, INF-γ, INF-β) were analyzed using the LEGENDplex™ Mouse Inflammation Panel (13-plex) in V-bottom plates (Biolegend, San Diego, CA, USA) and measured on a DxFlex cytometer (Beckman, Indianapolis, Indiana, USA) following the manufacturer’s instructions. Plasma concentration of adiponectin, corticosterone, and leptin (Crystal Chem, IL, USA), neutrophil elastase and MPO (R&D Systems, Inc., Minneapolis, USA) were measured using the ELISA method. The adapted ELISA was used to determine the MPO-DNA complex (33, 34). A 96-well plate (Sarstedt, Nümbrecht, Germany) was coated overnight at 4°C with 0.5 μg/mL anti-MPO antibody (0400-0002, Bio-Rad Laboratories, Hercules, CA, USA) in 0.1 M carbonate–bicarbonate buffer. 4% BSA (Merck, Saint Louis, MO, USA) in PBS (BR0095, Canvax Reagents SL, Valladolid, Spain) was used for blocking and sample dilution, while a 100-fold diluted anti-DNA POD antibody (11774425001, Roche, Basel, Switzerland) was applied for detection. The modified spectrophotometric method was used to determine concentrations of total cholesterol (CHOL) and triacylglycerol (TAG) in the liver (35, 36). Their accumulation was expressed in mmol per kg of liver tissue.

### 2.2 Statistical analyses

Before the experiment, the sample size was determined based on baseline body weight and 20% body weight gain (α= 0.05; P: 80%). Data were analyzed for normality with the Shapiro-Wilk normality test. Thus, data from inflammatory cytokines were log-transformed before statistical evaluation. Two-way ANOVA was performed separately for females and males, with independent factors of genotype and diet. Body weight dynamics and oGTT were evaluated using independent factors of time and group (genotype × diet), with a Bonferroni *post-hoc* correction in the JASP software. Graphs were created with GraphPad Prism version 8.0.1 (GraphPad Software, Inc., CA, USA). P-values less than 0.05 were considered significant. Detailed statistics are in the **Supplementary table**.

## 3 Results

### 3.1 Metabolic status in males

#### 3.1.1 Body weight, body composition, and caloric intake

**Figure 4:**
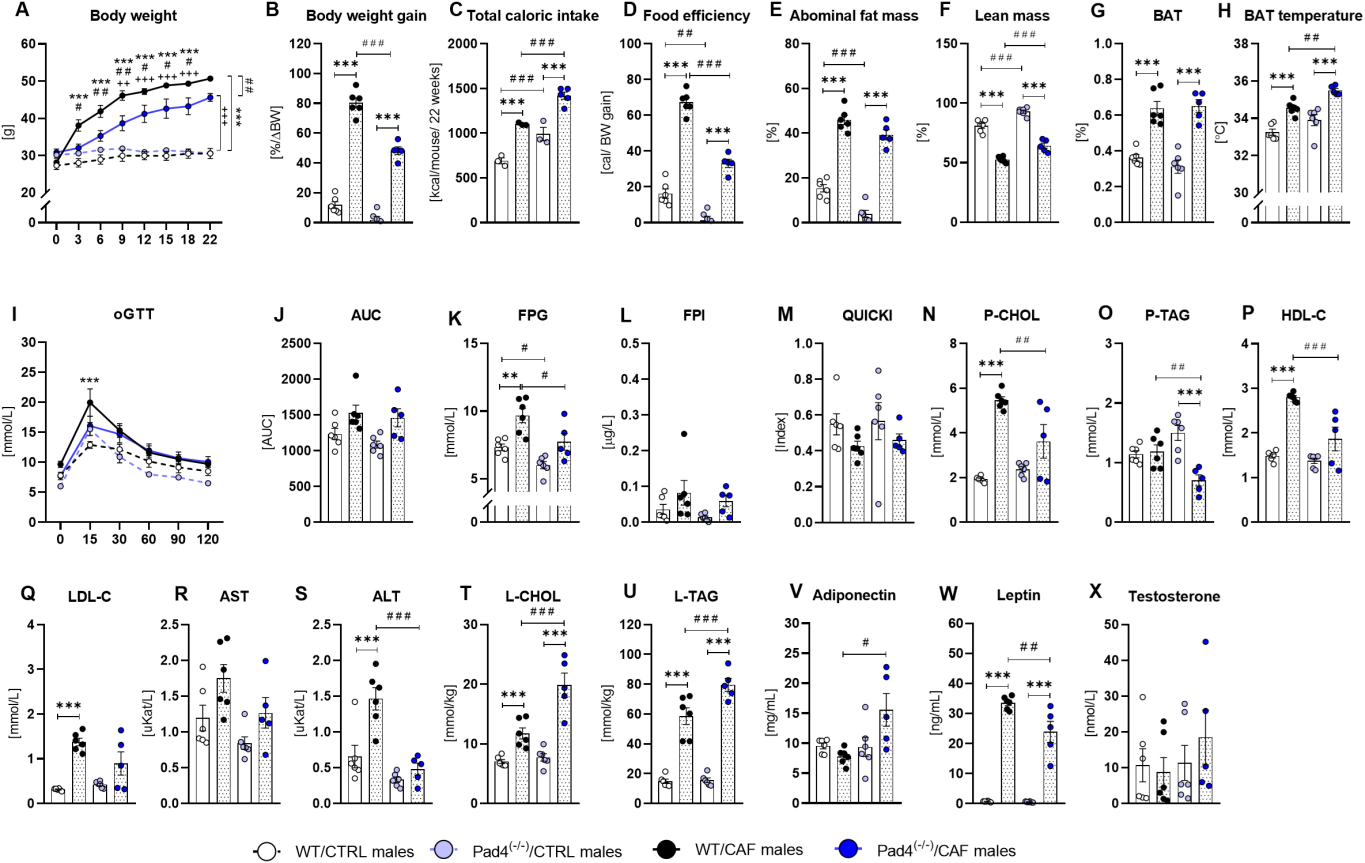
Metabolic status in Pad4^(-/-)^ and WT males. **(A)** Body weight during the 22 week-long cafeteria/control diet consumption; **(B)** Body weight gain; **(C)** Caloric intake in mice during obesogenic diet consumption; **(D)** Food efficiency; **(E)** Percentage of abdominal fat mass; **(F)** Total lean mass; **(G)** Percentage of brown fat (BAT); **(H)** Temperature of BAT; **(I)** Oral glucose tolerance test (oGTT); **(J)** Area under the glycemic curve (AUC); **(K)** Fasting plasma glycemia (FPG); **(L)** Fasting plasma insulin (FPI); **(M)** Quantitative insulin sensitivity check index (QUIKCI); **(N)** Cholesterol concentrations in plasma (P-CHOL); **(O)** Plasma triacylglycerol concentrations (P-TAG); **(P)** Plasma high-density lipoprotein cholesterol concentrations (P-HDL-C); **(Q)** Plasma low-density lipoprotein cholesterol concentrations (P-LDL-C); **(R)** Aspartate aminotransferase activity (AST); **(S)** Alanine aminotransferase activity (ALT); **(T)** Liver cholesterol content (L-CHOL); **(U)** Liver triacylglycerols content (L-TAG); **(V)** Adiponectin concentrations in plasma (**W**) Leptin concentrations in plasma (**X**) Plasma testosterone concentrations. WT/CTRL males: n=6; WT/CAF males: n=6; Pad4^(-/-)^/CTRL males: n=6; Pad4^(-/-)^/CAF males: n=5. Statistical analysis: **(A, I)** Repeated measures 2-way ANOVA with Bonferroni *post-hoc* test; **(B-H, J-X)** 2-way ANOVA with Bonferroni *post-hoc* test. Data are presented as the mean ± SEM. Statistical differences in: (A) ^*^WT/CAF vs. WT/CTRL; **^#^**Pad4^(-/-)^/CAF vs. Pad4^(-/-)^/CTRL; **^+^**Pad4^(-/-)^/CAF vs. WT/CAF. (B-R) ^*^CAF vs. CTRL (for both genotypes); **^#^**WT vs. Pad4^(-/-)^ (for both diet groups). **(A)**^#^p<0.05; ^++/##^p<0.01; ^***/###^p<0.001, **(B-R)**- ^*/#^p<0.05; ^**/##^p<0.01; ^***/###^p<0.001.

To evaluate the impact of PAD4 deficiency on diet-induced metabolic alterations, we first examined body weight, body composition, and caloric intake in male mice exposed to either a CTRL or CAF diet. Baseline mean body weights for six-month-old male mice were as follows: WT/CTRL (27.3 ± 2.6 g), WT/CAF (28.2 ± 1.6 g), Pad4^(-/-)^/CTRL (30.2 ± 2.2 g), Pad4^(-/-)^/CAF (30.8 ± 2.1 g). Two-way repeated measures of ANOVA showed a significant effect of time (p<0.001), diet (p<0.001), and their interaction (p<0.001; **Figure 4A**) on the body weight of mice (**Figure 4A**). According to *post-hoc* comparisons, WT/CAF males showed higher body weight in comparison to WT/CTRLs from the 3^rd^ week till the end of the experiment (all: p<0.05), while Pad4^(-/-)^/CAF showed higher body weight from the 9^th^ to the 22^nd^ week in comparison to Pad4^(-/-)^/CTRLs (all: p<0.05; **Figure 4A**). *Post-hoc* analysis also revealed that Pad4^(-/-)^/CAF males had significantly lower body weight compared than WT/CAF males throughout the experiment (all: p<0.05; **Figure 4A**). Consequently, the CAF diet-fed groups gained more weight compared to their controls (diet: p<0.001; WT/CTRL vs. WT/CAF: p<0.001; Pad4^(-/-)^/CTRL vs. Pad4^(-/-)^/CAF: p<0.001), while the PAD4 deficiency reduced body weight gain compared to WT mice under CAF diet conditions (genotype: p<0.001; diet x genotype interaction: p<0.001; **Figure 4B**).

Both CAF-diet groups consumed more calories than CTRL diet-fed mice (diet: p<0.001; **Figure 4C**). *Post-hoc* comparisons showed that WT/CAF males consumed on average 59.8% more calories (p<0.001) and Pad4^(-/-)^/CAF males by 71.4% more (p<0.001) that their respective controls (**Figure 4C**). In both dietary groups, Pad4^(-/-)^ mice consumed more calories than their WT peers (genotype: p<0.001; **Figure 4C**): Pad4^(-/-)^/CAF males by 18.3% (p<0.001) more calories than WT/CAF males, while Pad4^(-/-)^/CTRL males by 31.6% more calories than WT/CTRL males (p<0.001; **Figure 4C**). Regarding food preference, two-way ANOVA indicated genotype effect (p<0.01). Bonferroni *post-hoc* test showed that Pad4^(-/-)^/CAF males consumed fewer pellets and more processed food (p<0.01; **Supplementary Figure 1A**) and had higher fat intake (p<0.05) compared to WT/CAF males, while intakes of proteins and carbohydrates were similar (**Supplementary Figure 1B**).

Food efficiency was higher in both CAF diet-fed groups (diet: p<0.001) and also differed according to genotype (genotype: p<0.001; diet x genotype interaction: p<0.001; **Figure 4D**). *Post-hoc* analysis indicated that WT/CAF males showed higher food efficiency than WT/CTRLs (p<0.001) and Pad4^(-/-)^/CAF males higher than Pad4^(-/-)^/CTRLs (p<0.001; **Figure 4D**). Additionally, the Pad4^(-/-)^/CAF group showed lower food efficiency compared to WT/CAF males (p<0.001; **Figure 4D**). Both CAF diet-fed groups displayed higher abdominal fat mass than CTRLs (diet: p<0.001). *Post-hoc* results showed that WT/CAF males had 66.6% higher abdominal fat than WT/CTRL males (p<0.001), and Pad4^(-/-)^/CAF males by 90.1% higher compared with Pad4^(-/-)^/CTRLs (p<0.001; **Figure 4E**). Moreover, Pad4^(-/-)^/CTRL males showed 74.5% lower abdominal fat than WT/CTRL males (p<0.01; **Figure 4E**). Correspondingly, the percentage of lean mass was lower in both CAF diet-fed groups (diet: p<0.001): WT/CAF mice had 35% less lean mass than WT/CTRL mice (p<0.001), and Pad4^(-/-)^/CAF mice 30.9% less than Pad4^(-/-)^/CTRL mice (p<0.001; **Figure 4F**). Importantly, both Pad4^(-/-)^ groups showed a higher percentage of lean mass compared to WT groups (genotype: p<0.001), specifically, by 18% more in Pad4^(-/-)^/CAF mice compared to WT/CAFs (p<0.001), and by 13% more in Pad4^(-/-)^/CTRLs than WT/CTRL mice (p<0.001; **Figure 4F**). Regardless of genotype, two-way ANOVA revealed a significant effect of CAF diet (p<0.001) on brown adipose tissue (BAT) percentage. *Post-hoc* test indicated that Pad4^(-/-)^/CAF mice showed 79.4% more BAT than Pad4^(-/-)^/CTRLs (p<0.001), while WT/CAF mice differed from WT/CTRLs by 43.3% (p<0.001; **Figure 4G**).

In addition, to elucidate the impacts of PAD4 deficiency and CAF diet consumption on energy balance and tissue composition, we assessed BMC, BMD, and the temperature of BAT. A two-way ANOVA revealed the effect of diet (p<0.001) and genotype (p<0.01) on BMC. *Post-hoc* analysis confirmed higher BMC in both CAF groups of males in comparison to their CTRLs: WT/CAF vs. WT/CTRL (p<0.001); Pad4^(-/-)^/CAF vs. Pad4^(-/-)^/CTRL (p<0.001; **Supplementary Figure 1C**). Notably, the Pad4^(-/-)^/CAF males showed less BMC than WT/CAF males (p<0.05; **Supplementary Figure 1C**). BMD did not differ between diet groups while two-way ANOVA showed a main effect of genotype (p<0.01), though *post-hoc* tests failed to show between-group differences (**Supplementary Figure 1D**). The body temperature did not differ between groups of mice (**Supplementary Figure 1E**). BAT temperature differed according to diet (p<0.001) and genotype (p<0.001) factors. *Post-hoc* test indicated higher BAT temperature in both CAF groups in comparison to their CTRLs: WT/CAF vs. WT/CTRL (p<0.001); Pad4^(-/-)^/CAF vs. Pad4^(-/-)^/CTRL (p<0.001; **Figure 4H**). Additionally, Pad4^(-/-)^/CAF males showed higher BAT temperatures than WT/CAF males (p<0.01; **Figure 4H**). The consumption of a CAF diet led to a significant increase in body weight, fat mass, and caloric intake in male mice, while Pad4 deficiency reduced weight gain and improved lean mass and BAT temperature, suggesting a protective metabolic phenotype against CAF diet-induced obesity.

#### 3.1.2 Glucose metabolism

Subsequently, we evaluated glucose homeostasis to determine whether PAD4 deficiency affects CAF diet-induced alterations in glucose metabolism. Two-way ANOVA revealed a significant main effect of diet on FPG concentrations (p<0.001). *Post-hoc* tests indicated that WT/CAF mice had higher FPG than WT/CTRL mice (p<0.01; **Figure 4K**), while in Pad4^(-/-)^ males, no diet effects were present. Additionally, both Pad4^(-/-)^ groups of males showed lower FPG than WT diet-matched counterparts (genotype: p<0.001); Pad4^(-/-)^/CAF and WT/CAF groups (p<0.05; **Figure 4K**); Pad4^(-/-)^/CTRL and WT/CTRL groups (p<0.05; **Figure 4K**). The concentration of FPI was higher in CAF diet-fed mice (diet: p<0.05) and did not differ according to genotype. *Post-hoc* analysis did not detect significant differences between groups (**Figure 4L**). The QUICKI was lower in both CAF groups compared to genotype-matched CTRL-diet fed groups (diet: p<0.05), but the *post-hoc* analysis failed to identify differences (**Figure 4M**). The effect of genotype on the QUICKI was not observed (**Figure 4M**). Additionally, the dynamics of glycemia during the oral glucose tolerance test (oGTT) differed between the groups: a significant effect of group (p<0.001) on glycemia dynamics over time (time: p<0.001) was observed. *Post-hoc* analysis demonstrated that WT/CAF males showed a higher glycemic peak 15 minutes after glucose administration than WT/CTRL males (p<0.001; **Figure 4I**). The area under the glucose curve (AUC) was higher in the CAF groups in comparison to CTRLs (diet: p<0.01), with no effect of genotype or their interaction. *Post-hoc* analysis did not show any group differences (**Figure 4J**). The results demonstrate that the consumption of a CAF diet adversely affected glucose tolerance and elevated fasting glucose levels in male mice, but PAD4 deficiency did not significantly alter glucose metabolism under these conditions.

#### 3.1.3 Lipid metabolism

To further investigate systemic metabolic changes, we examined circulating lipid profiles in male mice. Both CAF groups showed higher plasma CHOL concentrations than genotype-matched CTRLs (diet: p<0.001), while a significant diet x genotype interaction was also observed (p<0.01). *Post-hoc* analysis showed significantly higher plasma CHOL concentrations in WT/CAF males compared to WT/CTRLs (p<0.001; **Figure 4N**). Additionally, the Pad4^(-/-)^/CAF mice showed lower concentrations of plasma CHOL compared to WT/CAFs (p<0.01; **Figure 4N**). Plasma TAG concentrations were affected by diet (p< 0.001) and diet x genotype interaction (p<0.001). *Post-hoc* analysis indicated lower plasma TAG concentrations in the Pad4^(-/-)^/CAF mice compared to the Pad4^(-/-)^/CTRL (p<0.001) and WT/CAF groups (p<0.01; **Figure 4O**). Plasma HDL-C concentrations were higher in both CAF diet-fed groups of mice (diet: p<0.001) and differed according to genotype (p<0.001) and their interaction (p<0.01). *Post-hoc* analysis confirmed that HDL-C concentrations were higher in WT/CAF than WT/CTRL mice (p<0.001; **Figure 4P**) but not in Pad4^(-/-)^ mice. Moreover, the Pad4^(-/-)^/CAF mice displayed lower circulating HDL-C concentrations than WT/CAF mice (p<0.001; **Figure 4P**). Both CAF diet-fed mouse groups had higher plasma LDL-C concentrations than CTRLs (diet: p<0.001), yet *post-hoc* analysis only showed a significant difference between WT/CAF and WT/CTRLs (p<0.001; **Figure 4Q**), with no genotype effect. In summary, consumption of the CAF diet resulted in higher plasma cholesterol and triglyceride concentrations, but Pad4 deficiency only partially mitigated these lipid changes, indicating that the absence of PAD4 regulates dyslipidemia.

#### 3.1.4 Liver-damage markers

To assess the potential hepatic consequences of CAF diet consumption and PAD4 deficiency, we measured circulating liver enzymes and hepatic lipid accumulation. Two-way ANOVA revealed a significant effect of diet (p<0.05) on AST activity, while no significant effects of genotype, or their interactions, were detected. *Post-hoc* test failed to show any group differences (**Figure 4R**). Additionally, both CAF groups showed higher ALT activities than their CTRL counterparts (diet: p<0.001) and differed according to the genotype (p<0.001) and interaction (p<0.05). The WT/CAF males displayed higher ALT activity than WT/CTRL males (p<0.001), but in Pad4^(-/-)^ mice, the *post-hoc* test did not show any statistical significance (**Figure 4S**). The Pad4^(-/-)^/CAF males compared to WT/CAF males showed reduced ALT activity (p<0.001; **Figure 4S**). The hepatic CHOL content was affected by (diet: p<0.001), genotype (p<0.001), and their interaction (p<0.01). *Post-hoc* analysis showed higher hepatic CHOL content in CAF diet-fed males: WT/CAF vs. WT/CTRL (p<0.001); Pad4^(-/-)^/CAF vs. Pad4^(-/-)^/CTRL (p<0.001; **Figure 4T**). Moreover, Pad4^(-/-)^/CAF mice showed higher hepatic CHOL content than WT/CAF mice (p<0.001; **Figure 4T**). Similarly, hepatic TAG content was affected by diet (p<0.001), genotype (p<0.01), and their interaction (p<0.05). Both CAF groups of males displayed higher hepatic TAG content than their CTRL counterparts: WT/CAF vs. WT/CTRL (p<0.001); Pad4^(-/-)^/CAF vs. Pad4^(-/-)^/CTRL (p<0.001; **Figure 4U**). Additionally, the Pad4^(-/-)^/CAF males showed higher hepatic TAG content than WT/CAF mice (p<0.001; **Figure 4U**). These results indicate that chronic consumption of a CAF diet results in lipid accumulation in the liver and elevated liver enzyme activity, characteristic of steatotic liver damage. PAD4 deficiency reduced ALT activity while elevating hepatic lipid accumulation, illustrating a complex role of PAD4 in liver metabolism during CAF diet consumption.

#### 3.1.5 Hormonal status

To deepen the characterization of metabolic changes, we examined circulating adipokines and sex hormones in male mice. Plasma adiponectin concentrations were influenced only by genotype (p<0.05) and the interaction of diet x genotype (p<0.05), but were not affected by diet factor. Circulating adiponectin was higher in the Pad4^(-/-)^/CAF males compared to the WT/CAF males (p<0.05; **Figure 4V**). For leptin, ANOVA showed a significant effect of diet (p<0.001), genotype (p<0.01), and their interaction (p<0.01). In both CAF groups, the leptin concentrations were higher compared to CTRL diet-fed mice: WT/CAF vs. WT/CTRL (p<0.001); Pad4^(-/-)^/CAF vs. Pad4^(-/-)^/CTRL (p<0.001; **Figure 4W**). Notably, lower concentrations of leptin were found in the Pad4^(-/-)^/CAF groups of males than in the WT/CAF males (p<0.01; **Figure 4W**). Plasma testosterone concentrations in males were not affected either by diet, genotype, or their interaction (**Figure 4X**). Corticosterone concentrations were slightly higher in both WT and Pad4^(-/-)^ males consuming CAF diet in comparison to genotype-matched controls, showing a significant main effect of diet (p<0.05), but not genotype on plasma corticosterone concentrations (**Supplementary Figure 1F**). The Pad4 deficiency in combination with CAF diet consumption led to higher circulating adiponectin and lower leptin concentrations, suggesting improved adipose tissue function despite high caloric intake. The observed CAF diet-induced increase in corticosterone concentrations supports activation of the hypothalamic–pituitary–adrenal axis under obesogenic conditions, which may contribute to the metabolic alterations seen in CAF diet-fed males.

### 3.2 Metabolic status in females

#### 3.2.1 Body weight, body composition, and caloric intake

**Figure 5:**
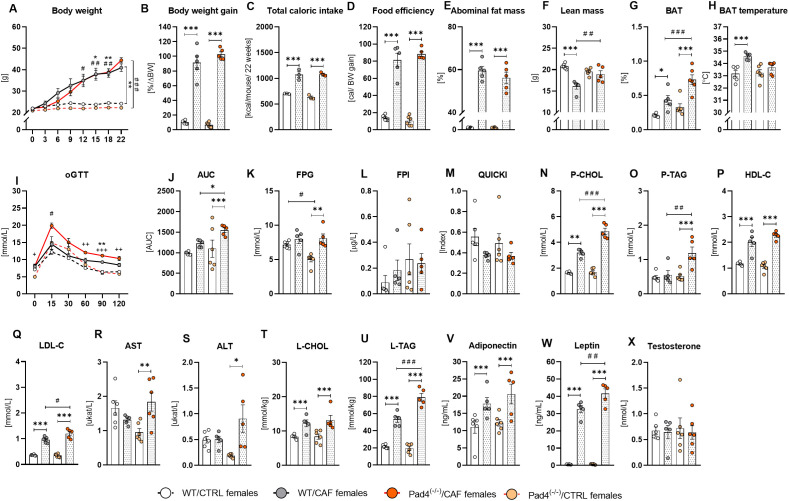
Metabolic status in Pad4^(-/-)^ and WT females. **(A)** Body weight during the 22 week-long cafeteria/control diet consumption; **(B)** Body weight gain; **(C)** Caloric intake in mice during obesogenic diet consumption; **(D)** Food efficiency; **(E)** Percentage of abdominal fat mass; **(F)** Total lean mass; **(G)** Percentage of brown fat (BAT); **(H)** Temperature of BAT; **(I)** Oral glucose tolerance test (oGTT); **(J)** Area under the glycemic curve (AUC); **(K)** Fasting plasma glycemia (FPG); **(L)** Fasting plasma insulin concentrations (FPI); **(M)** Quantitative insulin sensitivity check index (QUIKCI); **(N)** Cholesterol concentrations in plasma (P-CHOL); **(O)** Plasma triacylglycerol concentration (P-TAG); **(P)** Plasma high-density lipoprotein cholesterol concentration (P-HDL-C); **(Q)** Plasma low-density lipoprotein cholesterol concentrations (P-LDL-C); **(R)** Aspartate aminotransferase activity (AST); **(S)** Alanine aminotransferase activity (ALT); **(T)** Liver cholesterol content (L-CHOL); **(U)** Liver triacylglycerols content (L-TAG); **(V)** Adiponectin concentrations in plasma (**W**) Leptin concentrations in plasma (**X**) Plasma testosterone concentrations. WT/CTRL females: n=5; WT/CAF males: n=6; Pad4^(-/-)^/CTRL males: n=6; Pad4^(-/-)^/CAF males: n=5. Statistical analysis: **(A, I)** Repeated measures 2-way ANOVA with Bonferroni *post-hoc* test; **(B-H, J-X)** 2-way ANOVA with Bonferroni *post-hoc* test. Data are presented as the mean ± SEM. Statistical differences in: (A) ^*^WT/CAF vs. WT/CTRL; **^#^**Pad4^(-/-)^/CAF vs. Pad4^(-/-)^/CTRL. **^+^**Pad4^(-/-)^/CAF vs. WT/CAF (B-R) ^*^CAF vs. CTRL (for both genotypes); **^#^**WT vs. Pad4^(-/-)^ (for both diet groups). (A)^*/#^ p<0.05 ^**/##^ p<0.01, **(B-R)**- ^*/#/+^ p<0.05; ^**/##/++^ p<0.01; ^***/###/+++^ p<0.001.

To investigate potential sex-specific effects, we assessed body composition and caloric intake in female mice consuming CAF or control diets. Baseline mean body weights for six-month-old female mice were as follows: WT/CTRL (21.9 ± 1.3 g), WT/CAF (21.4 ± 0.5 g), Pad4^(-/-)^/CTRL (20.9 ± 1.2 g), Pad4^(-/-)^/CAF (21.9 ± 1.1 g). The body weight in females was affected by diet (p<0.001), time (p<0.001), and their interaction (p<0.001). Both CAF groups of females showed significantly higher body weight compared to genotype-matched controls from the 12^th^ week of CAF/CTRL diet consumption to the end of the experiment (all: p<0.05; **Figure 5A**). The genotype effects were not observed on the body weight of females. Mice fed with the CAF diet showed more significant body weight gain than control diet-fed females (diet: p<0.001): WT/CAF females gained 91.2% compared to 10.3% body weight gain in WT/CTRLs (p<0.001); Pad4^(-/-)^/CAF females gained 87.9% compared to 6.7% body weight gain in Pad4^(-/-)^/CTRLs (p<0.001; **Figure 5B**). Genotype differences in body weight gain were not present (**Figure 5B**).

Two-way ANOVA indicated the main effect of diet on caloric intake (p<0.001), without genotype or interaction effects. Both CAF diet-fed groups of females consumed more calories than controls: WT/CAF vs. WT/CTRL (p<0.001); Pad4^(-/-)^/CAF vs. Pad4^(-/-)^/CTRL (p<0.001; **Figure 5C**). Despite no genotype differences in the caloric intake (**Figure 5C**), Pad4^(-/-)^/CAF females preferred more processed food (p<0.001) and less salty food (p<0.01) resulting in higher consumption of fat (p<0.01) and less carbohydrate intake than WT/CAF females (p<0.001; **Supplementary Figures 1G, H**). The ANOVA showed significant main effects of diet (p<0.001) on food efficiency, with no genotype effect or effect of their interaction. Both CAF groups of females showed higher food efficiency: in WT/CAF females, it was 5.8-fold higher than WT/CTRLs (p<0.001); while Pad4^(-/-)^/CAF females showed 7.2-fold higher food efficiency than Pad4^(-/-)^/CTRLs (p<0.001; **Figure 5D**).

The two-way ANOVA showed that both CAF groups had more abdominal fat mass (diet: p<0.001), with no genotype or genotype x diet interaction (**Figure 5E**). The WT/CAF females showed higher fat mass than WT/CTRLs (p<0.001), and Pad4^(-/-)^/CAF females than Pad4^(-/-)^/CTRLs (p<0.001; **Figure 5E**). Both CAF diet-fed groups showed less lean mass compared to their CTRLs (diet: p<0.001): WT/CAF mice had lower lean mass than WT/CTRLs (p<0.001) and Pad4^(-/-)^/CAF mice than Pad4^(-/-)^/CTRLs (p<0.001; **Figure 5F**). ANOVA showed a main effect of diet (p<0.001) on BAT percentage, with an additional significant effect of genotype (p<0.001) and diet x genotype interaction (p<0.05). Females consuming the CAF diet showed a greater percentage of the BAT than CTRL diet-fed mice: the WT/CAF group showed more BAT than WT/CTRLs (p<0.05), and Pad4^(-/-)^/CAF mice displayed more BAT than Pad4^(-/-)^/CTRLs (p<0.001; **Figure 5G**). Notably, Pad4^(-/-)^/CAF females showed a higher BAT percentage than WT/CAF females (p<0.001; **Figure 5G**). Overall, in females, long-term CAF diet consumption resulted in higher body weight and fat mass, while Pad4 deficiency had no effects on these variables.

In addition, in females, we investigated the possible effects of PAD4 deficiency and long-term CAF diet consumption on skeletal and thermogenic parameters by evaluating bone mineral content and density and the temperature of the body, as well as brown adipose tissue. ANOVA indicated a main effect of diet (p<0.001) on BMC, but no effect of genotype or interactions of these factors. Both CAF diet-fed groups of females showed higher BMC compared to CTRLs: WT/CAF vs. WT/CTRL (p<0.001); Pad4^(-/-)^/CAF vs. Pad4^(-/-)^/CTRL (p<0.001; **Supplementary Figure 1I**). For BMD, ANOVA showed significant main effects of diet (p<0.01) and genotype (p<0.05), without their interaction. The BMD was higher in CAF females than in CTRLs, while the *post-hoc* test showed significantly higher BMD only in the WT/CAF females in comparison to WT/CTRLs (p<0.05; **Supplementary Figure 1J**). On the contrary, ANOVA revealed a significant effect of genotype (p<0.001) on body temperature but no effect of diet or interaction of these factors. *Post-hoc* analysis confirmed higher body temperature in Pad4^(-/-)^/CTRLs compared to WT/CTRLs (p<0.05), with no difference between CAF diet-fed groups (**Supplementary Figure 1K**). For BAT temperature, ANOVA showed a significant effect of diet (p<0.001) and diet x genotype interaction (p<0.05), while the effect of genotype was not significant. WT/CAF females displayed higher BAT temperature than WT/CTRLs (p<0.001), while Pad4^(-/-)^/CAF females did not differ from Pad4^(-/-)^/CTRLs. However, the Pad4^(-/-)^/CAF females tended to show lower BAT temperature than WT/CAF females (p=0.06; **Figure 5H**). These findings indicate that chronic consumption of the CAF diet elevates bone mineral content and brown adipose tissue temperature in females, whereas PAD4 deficiency is associated with a slight decrease in bone mineral density and a tendency for reduced BAT thermogenic activity. Therefore, PAD4 may play a role in preserving bone and energy balance in females under high-caloric dietary conditions.

#### 3.2.2 Glucose metabolism

We subsequently investigated glucose metabolism in female mice to determine whether PAD4 deficiency affects glucose homeostasis. The FPG was affected by diet (p<0.001), genotype (p<0.05) factors and their interaction (p<0.05). *Post-hoc* analysis showed that FPG was higher in the Pad4^(-/-)^/CAF mice compared to the Pad4^(-/-)^/CTRLs (p<0.01; **Figure 5K**). Additionally, Pad4^(-/-)^/CTRL females showed lower FPG than WT/CTRLs (p<0.05; **Figure 5K**). No significant difference was found between WT/CAF and Pad4^(-/-)^/CAF groups. The FPI did not differ according to diet or genotype factors (**Figure 5L**), while QUIKCI was lower in the CAF diet-fed mice (diet: p<0.05), with no group differences in *post-hoc* analysis (**Figure 5M**). For the oGTT, ANOVA indicated a significant effect of group (p<0.001), time (p<0.001), and their interaction (p<0.01). *Post-hoc* comparisons revealed that WT/CAF females exhibited higher glycemia at 90 minutes after glucose load than WT/CTRLs (p<0.05; **Figure 5I**). Significantly higher glycemia in Pad4^(-/-)^/CAF females compared to Pad4^(-/-)^/CTRL females was observed at 60 (p<0.01), 90 (p<0.001), and 120 minutes (p<0.01) after glucose load. Additionally, Pad4^(-/-)^/CAF females displayed higher glycemia than WT/CAF females at 15 minutes (p<0.05), indicating an early post-load impairment (**Figure 5I**). ANOVA confirmed a significant main effect of diet (p<0.001) and genotype (p<0.01) on the glycemic AUC. *Post-hoc* analysis demonstrated that the AUC was higher in Pad4^(-/-)^/CAF females compared to Pad4^(-/-)^/CTRLs (p<0.001) and WT/CAF females (p<0.05; **Figure 5J**). These results suggest that a CAF diet negatively affects glucose tolerance in females, particularly in Pad4^(-/-)^ mice, indicating that PAD4 may have a sex-dependent impact on maintaining glucose homeostasis.

#### 3.2.3 Lipid metabolism

Next, we measured plasma lipid markers to determine the impact of PAD4 deficiency on lipid metabolism in females. Plasma total CHOL concentrations were affected by diet (p<0.001), genotype (p<0.001), and their interaction (p<0.001). Both CAF diet-fed groups of females showed higher plasma CHOL concentrations than the genotype-matched CTRLs: WT/CAF vs. WT/CTRL females (p<0.01), Pad4^(-/-)^/CAF vs. Pad4^(-/-)^/CTRL females (p<0.001), while Pad4^(-/-)^/CAF showed higher plasma cholesterol concentrations than WT/CAFs (p<0.001; **Figure 5N**). Significant effects of CAF diet consumption (p<0.01), as well as genotype (p<0.01), and a diet x genotype interaction (p<0.05) were found on circulating TAG concentrations. *Post-hoc* comparisons revealed that Pad4^(-/-)^/CAF females displayed higher plasma TAG concentrations than Pad4^(-/-)^/CTRLs (p<0.001) and WT/CAFs (p<0.01; **Figure 5O**). For plasma HDL-C concentrations, ANOVA demonstrated only a diet effect (p<0.001) with higher circulating HDL-C concentrations in CAF diet-fed females: WT/CAF vs. WT/CTRL (p<0.001), Pad4^(-/-)^/CAF vs. Pad4^(-/-)^/CTRL (p<0.001; **Figure 5P**). Plasma LDL-C concentrations were affected by diet (p<0.001), genotype (p<0.05) and their interaction (p<0.05). Plasma LDL-C concentrations were higher in both CAF diet-fed groups: WT/CAF vs. WT/CTRL (p<0.001), Pad4^(-/-)^/CAF vs. Pad4^(-/-)^/CTRL (p<0.001), while higher LDL-C levels were in Pad4^(-/-)^/CAF compared to WT/CAFs (p<0.05; **Figure 5Q**). In females, long-term consumption of the CAF diet increased plasma lipid concentrations regardless of genotype. However, Pad4^(-/-)^/CAF females exhibited elevated cholesterol, triglycerides, and LDL-C, suggesting a possible dysregulation of lipid metabolism due to the absence of PAD4.

#### 3.2.4 Liver-damage markers

To evaluate potential hepatic effects of chronic CAF diet consumption and PAD4 deficiency in females, we measured plasma liver enzymes and hepatic lipid accumulation. According to two-way ANOVA, there was no significant effect of diet or genotype, just their interaction (p<0.05) on plasma AST activity, which was higher in the Pad4^(-/-)^/CAF females compared to Pad4^(-/-)^/CTRLs (p<0.01; **Figure 5R**), while there were no differences in WT mice. Similarly, ANOVA showed a significant effect of diet (p<0.05) and diet x genotype interaction (p<0.05) on plasma ALT activity. The *post-hoc* test confirmed that Pad4^(-/-)^/CAF females showed higher activity of ALT in plasma compared to Pad4^(-/-)^/CTRL females (p<0.05; **Figure 5S**). Two-way ANOVA further indicated a main effect of diet on hepatic CHOL accumulation (p<0.001), with no genotype or interaction effects: both CAF diet-fed groups exhibited higher hepatic CHOL content than their respective controls (WT/CAF vs. WT/CTRL (p<0.001); Pad4^(-/-)^/CAF vs. Pad4^(-/-)^/CTRL (p<0.001; **Figure 5T**). Both CAF groups of females showed higher hepatic TAG content compared to genotype-matched CTRLs (diet: p<0.001, genotype: p<0.001; interaction: p<0.001): WT/CAF vs. WT/CTRL (p<0.001); Pad4^(-/-)^/CAF vs. Pad4^(-/-)^/CTRL (p<0.001; **Figure 5U**). Notably, the Pad4^(-/-)^/CAF group of females showed higher hepatic TAG content than the WT/CAF females (**Figure 5U**). Accordingly, in females, long-term CAF diet consumption increased circulating lipid concentrations regardless of genotype. However, Pad4^(-/-)^/CAF females exhibited higher triglycerides, indicating possible dysregulation of lipid metabolism in the absence of PAD4.

#### 3.2.5 Hormonal status

Next, we evaluated endocrine responses to CAF diet intake and PAD4 deficiency in females by analyzing plasma adipokine and sex hormone concentrations. Plasma adiponectin concentrations were affected only by the diet factor (p<0.001), where *post-hoc* analysis confirmed that both CAF-fed groups exhibited higher plasma adiponectin concentrations compared to genotype-matched controls: WT/CAF vs. WT/CTRL (p<0.001), and Pad4^(-/-)^/CAF vs. Pad4^(-/-)^/CTRL (p<0.001; **Figure 5V**). For plasma leptin, ANOVA indicated the effect of diet (p<0.001), genotype (p<0.01), and their interaction (p<0.01). *Post-hoc* comparisons revealed that both CAF groups showed higher plasma leptin concentrations than their respective controls: WT/CAF vs. WT/CTRL (p<0.001); Pad4^(-/-)^/CAF vs. Pad4^(-/-)^/CTRL (p<0.001; **Figure 5W**). In addition, higher concentrations of leptin were present in the Pad4^(-/-)^/CAF females compared to the WT/CAF females (p<0.01; **Figure 5W**). Two-way ANOVA showed no significant effects of diet, genotype, or their interaction on plasma testosterone concentrations (**Figure 5X**). Corticosterone concentrations were affected only by the diet x genotype interaction (p<0.05), where Pad4^(-/-)^/CAF females had higher corticosterone concentrations compared to both WT/CAF (p<0.05) and Pad4^(-/-)^/CTRL females (p<0.05; **Supplementary Figure 1L**). Consequently, our results demonstrate that long-term consumption of a CAF diet elevates plasma adipokine concentrations in females, regardless of genotype. In Pad4^(-/-)^/CAF females, higher leptin concentrations have been found, indicating increased fat mass and altered adipose tissue regulation. Moreover, the elevated corticosterone concentrations in Pad4^(-/-)^/CAF females suggest enhanced hypothalamic–pituitary–adrenal axis activity under obesogenic conditions, which may contribute to the sex-specific metabolic and endocrine alterations related to PAD4 deficiency.

### 3.3 Home-cage monitoring

To assess potential sex-dependent behavioral (locomotor activity, sleep behavior) and metabolic effects (food and water intake) of chronic CAF diet consumption and PAD deficiency, both female and male mice were subjected to 24-hour PhenoTyper-cage monitoring.

#### 3.3.1 Males

The locomotor activity of WT/CAF and Pad4^(-/-)^/CAF males did not differ during the 24-hour testing period (**Figure 2A**). The Pad4^(-/-)^/CAF males showed an earlier movement onset before the active phase than WT/CAF males (**Figure 2A**). The Pad4 deficiency did not affect the circadian rhythm of food intake and was comparable to food intake of WT/CAF males in both light and dark phase (**Figure 2B**). Moreover, caloric intake during the 24-hour PhenoTyper-cage monitoring was similar in both male CAF groups (**Figure 2B**). While WT/CAF males showed continuous sleeping periods, the Pad4^(-/-)^/CAF males showed more frequent sleep disruptions during the light phase (**Figure 2C**). During the light phase, the AUC of sleeping time did not differ between Pad4^(-/-)^/CAF and WT/CAF males (**Figure 2C**). Additionally, Pad4^(-/-)^/CAF males showed higher water intake during the light phase (p<0.05), but not in the dark phase, compared to WT/CAF males (**Figure 2D**). These results suggest that PAD4 deficiency had no significant impact on overall locomotor activity or food intake in CAF-fed males. However, it was linked to an earlier onset of activity and disrupted sleep throughout the light phase of the light/dark cycle.

#### 3.3.2 Females

Pad4^(-/-)^/CAF females showed earlier movement onset before the active phase than WT/CAF females, resulting in higher locomotor activity in Pad4^(-/-)^/CAF females during the dark phase compared to WT/CAF females (p<0.05; **Figure 2E**). The Pad4^(-/-)^/CAF females showed similar food intake patterns during light phase, with a non-significant increase in dark phase, compared to the WT/CAF females. The overall 24-hour caloric intake was higher in Pad4^(-/-)^/CAF females compared to WT/CAF females (p<0.05; **Figure 2F**). The WT/CAF females showed more disrupted sleep compared to the Pad4^(-/-)^/CAF females in the light phase (p<0.05), but not in the dark phase (**Figure 2G**). Pad4^(-/-)^/CAF females did not differ in water consumption from WT/CAF females during either the light or dark phases (**Figure 2H**). In CAF-diet-fed females, PAD4 deficiency was associated with higher locomotor activity and caloric consumption, together with greater quality of sleep, suggesting unique sex-specific behavioral responses to CAF diet consumption.

### 3.4 NETs markers and inflammation

To examine the effect of PAD4 deficiency on NET formation and inflammation, circulating NET markers and mediators of inflammation were assessed in both males and females.

#### 3.4.1 Males

Regarding neutrophils, two-way ANOVA indicated only genotype effect (p<0.001): neutrophil counts were higher in Pad4^(-/-)^ mice compared to their WT counterparts in both dietary groups, although the pairwise post- hoc tests did not reach statistical significance (**Figure 3C**). ANOVA revealed only genotype effect (p<0.001) on the percentage of NETotic PMNs. *Post-hoc* comparisons demonstrated that the percentage of CitH3^+^ ecDNA^+^ NETotic neutrophils was lower in both Pad4^(-/-)^ groups compared to their WT counterparts: Pad4^(-/-)^/CAF males showed significantly lower NETs percentages than WT/CAF males (p<0.001), and Pad4^(-/-)^/CTRLs than WT/CTRLs (p<0.001). Consumption of the CAF diet did not affect the percentage of NETotic PMNs (**Figure 3D**). The amount of the MPO-DNA complexes did not differ according to the diet consumed or genotype of mice (**Figure 3E**). For circulating ecDNA, ANOVA indicated a diet effect (p<0.05), but *post-hoc* analysis failed to reveal any significant pairwise differences between groups. Plasma MPO concentrations did not differ according to genotypes, but a significant effect of diet (p<0.05) was observed, where WT/CAF males showed higher concentrations of MPO than WT/CTRL males (p<0.05; **Figure 3G**). The concentration of neutrophil elastase in plasma was not affected by long-term CAF diet consumption, however, Pad4^(-/-)^/CTRL mice showed higher concentrations of neutrophil elastase than WT/CTRLs (genotype: p<0.05; **Figure 3H**). DNase activity was not affected by CAF diet consumption, however, the effect of genotype (p<0.05) was significant: Pad4^(-/-)^/CAF mice showed lower DNase activity than WT/CAF mice (p<0.05; **Figure 3I**). The concentrations of inflammatory cytokines and chemokines did not differ between groups of mice (**Supplementary Figure 2A**). These observations support the expected decrease in NET production in Pad4^(-/-)^ males. These results indicate that long-term CAF diet consumption did not significantly affect systemic inflammation in WT and Pad4^(-/-)^ males.

#### 3.4.2 Females

In females, the number of neutrophils was affected only by genotype of mice (p<0.01). *Post-hoc* analysis showed that both Pad4^(-/-)^ groups displayed higher neutrophil counts in blood than their WT female counterparts (**Figure 3J**), while statistical significance was reached only in CTRL diet-fed females (Pad4^(-/-)^/CTRL vs. WT/CTRL females, p<0.05; **Figure 3J**). For the percentage of circulating NETotic PMNs, ANOVA revealed only the effect of genotype (p<0.001). Circulating NETotic PMNs were lower in the Pad4^(-/-)^ mice than in WT counterparts: Pad4^(-/-)^/CAF vs. WT/CAF females (p<0.05); Pad4^(-/-)^/CTRL vs. WT/CTRL females (p<0.01; **Figure 3K**). Two-way ANOVA indicated no significant effects of diet, genotype, or their interaction for MPO–DNA complex formation, circulating ecDNA concentration, or DNase activity (**Figures 3L, M, P**). For plasma MPO concentrations, ANOVA revealed only a significant effect of diet (p<0.001). *Post-hoc* analysis demonstrated that both CAF-fed groups of females had higher plasma MPO concentrations than their genotype-matched controls: WT/CAF vs. WT/CTRL (p<0.001) and Pad4^(-/-)^/CAF vs. Pad4^(-/-)^/CTRL (p<0.05; **Figure 3N**). Plasma neutrophil elastase concentrations were affected by both diet (p<0.001), genotype (p<0.05) and their interaction (p<0.01). Bonferroni *post-hoc* test showed that WT/CAF females had higher plasma neutrophil elastase concentrations than both Pad4^(-/-)^/CAF (p<0.01) and WT/CTRL females (p<0.001; **Figure 3O**). Circulating inflammatory cytokines and chemokines did not differ according to diet or genotype of female mice (**Supplementary Figure 2B**). Similarly to males, PAD4 deficiency substantially reduced the quantity of circulating NETotic neutrophils in females. While the long-term consumption of a CAF diet appeared not to markedly increase systemic inflammatory cytokine concentrations, the diet-specific markers of neutrophil activation (neutrophil elastase and myeloperoxidase) were observed, which need to be further investigated.

## 4 Discussion

The Pad4^(-/-)^ mice of both sexes, regardless of whether they consumed a standard or an obesogenic CAF diet, displayed higher neutrophil counts in peripheral blood and, as expected, a lower percentage of circulating PAD4-derived NETs than their WT counterparts. Here we show that the manifestation of adiposity and obesity-associated dysmetabolic features in Pad4^(-/-)^ mice is sex- and diet-specific.

PAD4, a key enzyme in the induction of NET release, is predominantly expressed in hematopoietic cells (37). Adipocytes, cells of the exocrine or endocrine pancreas, or hepatocytes do not express PAD4 mRNA or PAD4 protein (38). Thus, the localization of PAD4 within tissues that manifest obesity-associated dysmetabolism, such as adipose tissue, liver, or pancreas, reflects the recruitment of neutrophils (or macrophages) in response to metabolic challenges. Our full-body Pad4^(-/-)^ mice produced negligible amounts of NETs through histone citrullination, which aligns with other studies in mice with specific knockout of PAD4 (18, 39) or pharmacological PAD4 inhibition (17, 40, 41). Our finding of similar quantities of MPO-DNA complexes across all groups confirms that PAD4 deletion does not entirely prevent NET formation (42). Plasma ecDNA levels also did not differ between genotypes, sexes, or dietary groups. The elevated neutrophil counts observed in Pad4^(-/-)^ mice, together with higher neutrophil elastase and MPO levels, may indicate a shift in hematopoiesis and neutrophil functional adaptation, which might be sex specific. Since data on the effects of PAD4 deletion or its pharmacological inhibition under a CAF diet are not available, we compared our results with those obtained under an HFD. The floxed male mice on the HFD and those with deleted PAD4 in hematopoietic cells (Padi4KO) also exhibited similar levels of plasma double-strand DNA, despite NETosis being effectively blocked in the knockouts (39). Thus, the determination of total ecDNA or unspecific MPO-DNA complexes for citrullination does not provide information on the contribution of PAD4-derived NETs. It also remains unclear to what extent NET-derived DNA contributes to plasma ecDNA concentrations. Autologous ecDNA originates from the passive release of nuclear and mitochondrial DNA from dying cells, as well as the active secretion of DNA via NETosis, the production of exosomes, and the extracellular release of vesicles (43). DNA in NETs is structurally more accessible and thus more rapidly degraded by DNase I than ecDNA derived from apoptosis or necrosis (44). Short fragments of ecDNA are rapidly eliminated by macrophages and renal clearance. In standard DNA quantification methods, they are often underrepresented or may entirely escape detection (43, 45). We also need to consider that the quantification of NETs in plasma is not indicative of the degree of NETosis in tissues (46).

In healthy subjects, circulating ecDNA concentrations show a direct relationship with body mass index or visceral fat area (47). Individuals with obesity exhibit higher plasma and adipose tissue levels of MPO-DNA complexes than eutrophic controls (12, 46). The highest proportion of circulating ecDNA in patients with obesity originates from blood-derived and hematopoietic cells (approximately 22%). Visceral adipocytes, similar to hepatocytes, and cells derived from the pancreas and nervous system, each account for 6% to 8% of the total ecDNA (48). Regardless of phenotype and sex, our obese and lean mice exhibited similar ecDNA concentrations, suggesting that ecDNA concentrations are not related to obesity.

The onset of obesity differs between women and men. Before menopause, men are generally more affected by obesity, however, after this critical age, the prevalence is reversed with a higher incidence observed in women (23). In mouse models, female mice show delayed obesity progression than males after 10 weeks (49) or 17 weeks (50) of HFD consumption. Obesity-associated hyperglycemia and insulin resistance also show sex-specific patterns. Females fed with HFD display less severe impairments in insulin sensitivity, hyperglycemia, and overall glycemic control than males after 10 weeks (49), and even more markedly after 35 weeks of HFD diet consumption (51). Whether these sex-specific effects are related to the protective role of estrogens or to sex-dependent differences in energy metabolism under obesogenic conditions requires further investigation.

As assumed, PAD4 deficiency did not affect body weight gain in mice of either sex on a standard diet. However, in males, it beneficially affected central fat distribution, and to gain similar weight as their WT counterparts, Pad4^(-/-)^ males consumed about 30% more calories. Under the obesogenic CAF diet, PAD4 deficiency delayed and ameliorated body weight gain, and was associated with increased BAT mass and BAT temperature. The induction of thermogenesis via activation of uncoupling protein 1 (UCP-1) biosynthesis in BAT is the primary mechanism leading to lower weight gain in males consuming a CAF diet (52, 53). We were unable to analyze the expression of UCP-1 and explore the downstream pathways in BAT due to technical reasons. However, under PAD4 deletion, a higher BAT temperature indirectly suggests modulation of energy expenditure via UCP-1. In contrast to males, PAD4 deletion did not affect body weight gain or caloric intake in female mice consuming the CAF diet. Despite the higher BAT mass, the thermogenic effect was not observed. Our data support the previous findings that the adaptive mechanisms regulating energy balance are sex- and diet-specific; for example, under an HFD condition, thermogenic response is manifested only in female rodents (54). Further research is needed to elucidate how PAD4 deficiency amplifies the thermogenic effects of the CAF diet in males. Chronic overeating is associated with metabolic stress, which might induce corticosterone release and contribute to obesity (55). Following long-term CAF diet consumption Pad4^(-/-)^ females showed high corticosterone levels, however, Pad4^(-/-)^ males not. Whether Pad4 deficiency or altered NETosis contributes to more severe obesity in a sex-specific manner remains to be investigated. Studies comparing the obesogenic effects of a traditional high-fat lard-based diet versus a CAF diet in male mice (56) or rats (57) show that a CAF diet intake induces higher body, white, and brown adipose tissue weight, higher cholesterolemia, more severe organ damage (including liver steatosis), and increased systemic and tissue inflammation. The effects on glucose intolerance and insulin resistance are similar. In the HFD-induced obesity model in male mice, deletion of PAD4 in hematopoietic cells (Padi4KO) or pharmacologic inhibition of PADI4 by the selective PADI4 inhibitor GSK484 did not affect body weight gain or fat accumulation (39). After 5 weeks of intervention with an HFD, male mice with neutrophil-selective deletion of PAD4 showed lower body weight than the WT animals. In a different cohort consuming an HFD for 10 weeks, no significant differences were recorded in either sex (18). In mice fed an HFD for 10 weeks, either the C57BL/6J (40) or apolipoprotein-E deficient (41), PAD4 blockade with Cl-amidine did not affect body weight gain (the sex of the mice was not specified). Whether the trend towards higher locomotor activity observed in our Pad4^(-/-)^ CAF males during home cage monitoring could contribute to higher energy expenditure in the long term remains unclear. As testosterone levels were not affected by PAD4 deletion or the CAF diet intake in either sex, the observed changes in adiposity were testosterone independent. Thus, the differences in body weight gain between our study and the aforementioned ones reflect sex differences in response to different dietary interventions in Pad4^(-/-)^ animals. Moreover, the age of onset of obesity may have played a role (58): studies on the effects of an HFD were initiated in adolescent mice, whereas we started the CAF diet in adult mice.

In patients with obesity, the amounts of plasma ecDNA or MPO-DNA complexes correlate directly with measures of adiposity, markers of inflammation, glucose, and lipid metabolism (12, 46, 48), suggesting that NETosis may represent a link between overfeeding and obesity-associated dysmetabolism. In our study, deletion of PAD4 did not affect insulin sensitivity (assessed by the QUICKI) in either sex, but female Pad4^(-/-)^ mice on a CAF diet were less glucose tolerant. This sex difference could be at least partially attributed to a compensatory increase in adiponectinemia in Pad4^(-/-)^ males on the CAF diet. In male mice administered an HFD, deletion of *PADi4* in hematopoietic cells improved glucose tolerance and insulin sensitivity (determined using a HOMA index) (39), while deletion of PAD4 in neutrophils resulted in lower glycemia (18) compared with controls. Pharmacological inhibition of PAD4 with Cl-amidine in C57BL/6J mice on the HFD did not affect glucose tolerance or insulin resistance (40). In contrast, in female non-obese diabetic mice, it improved fasting glycemia, enhanced glucose tolerance, and delayed the onset and incidence of type 1 diabetes mellitus (T1DM) (17). In Pad4^(-/-)^ animals, we also observed sex differences in lipid metabolism. PAD4 deficiency rescued males on the CAF diet from a surge in cholesterolemia and triacylglycerolemia, but the animals accumulated higher amounts in the liver. In females, cholesterol levels remained unaffected, while TAG accumulated in plasma and liver. Under an HFD, neither knocking out Padi4 nor pharmacological inhibition of PAD4 via GSK484 administration affected plasma lipid levels, but it did blunt liver steatosis (39). PAD4 blockade with Cl-amidine did not significantly affect plasma lipid profile either in C57BL/6J (40) or in Apoe^(−/−)^ mice (41) on an HFD. Recently, we showed that 10 weeks on the CAF diet in female mice induced non-fibrotic steatosis, increased hepatic TAG and CHOL storage, and led to the presence of NETs in the liver, while NETs were associated with the severity of steatosis (59). Whether the manifestation of steatosis in Pad4^(-/-)^ mice is linked to the CAF diet model itself, to impaired hepatic lipid clearance, or to direct effects of the whole-body PAD4 deficiency on hepatocyte physiology remains to be elucidated.

According to Albiero et al. (39), the driver of chronic inflammation in HFD-induced obesity is gut dysbiosis, which induces PAD4-mediated NETosis at the interface between the microbiota and the intestine, thereby disrupting the gut epithelial barrier’s integrity. The resulting spillover of bacterial products from the gut elicits metabolic endotoxemia. Abrogating NETosis through the genetic deletion of PAD4 or its pharmacological inhibition protects mice from intestinal hyperpermeability and the propagation of systemic inflammation, ultimately leading to dysmetabolism. As shown in male rats, the CAF diet also alters gut microbiota diversity and phylum abundance, which is associated with endotoxemia, markers of obesity, glucose, and lipid metabolism (60). However, the CAF diet is a high-fat, high-carbohydrate diet, distinct from the HFD in terms of its palatability, variety, and novelty, which drives voluntary hyperphagia. These factors may underlie the different metabolic outcomes and sex dimorphism observed in the PAD4 deletion-induced obesity phenotype on a CAF diet.

### 4.1 Strengths and limitations

To our knowledge, we provide the first data on the role of Pad4 deficiency in CAF diet-induced obesity and obesity-mediated metabolic and behavioral changes in mice of both sexes, with obesity initiated in adult animals. However, this study has several limitations. Although we measured a wide panel of inflammatory markers in plasma, high technical variability prevented us from drawing definitive conclusions. Due to technical constraints, we do not provide data on the histopathology of fat and liver tissue, or immunohistological analysis of NETs in the liver. However, we recently demonstrated that after CAF diet consumption, the accumulation of neutrophils and NETs markers is present (59). Moreover, we did not analyze gene or protein expression related to thermoregulation, indirect calorimetry to quantify energy expenditure, or microbiome composition. We acknowledge that hormonal fluctuations across the estrous cycle may contribute to variability in some parameters. However, estrous staging was not performed to avoid repeated handling stress, which could influence behavioral and metabolic outcomes in this experiment.

## 5 Conclusion

We demonstrated that in the model of CAF diet-induced obesity in adult mice, a full-body knockout of PAD4 has, on the one hand, a sex-specific effect on the onset of obesity and its complications. On the other hand, it might lead to unintended consequences, such as liver steatosis, in both sexes. This confirms that PAD4’s contribution to obesity-associated complications and their progression is not limited to, but extends beyond, its enzymatic activity in the formation of NETs. To obtain a comprehensive understanding of the effects of Pad4 deficiency in diet-induced models of obesity, future studies should address the outcomes of different obesogenic diets (e.g., the high saccharide diet), the effects of age (e.g., initiation of obesity in prepubertal, adolescent, and old animals), and sex differences. Investigating the exact molecular mechanisms by which NETs exert their effects is crucial for a complete understanding of their pathological impact and for the development of targeted PAD4 inhibitors or NET-modulating therapies leading to effective and safe treatments for the multifaceted challenges posed by obesity-associated metabolic disorders.

## Data Availability

The datasets presented in this study can be found in online repositories. The names of the repository/repositories and accession number(s) can be found in the article/**Supplementary Material**.
