# Supplementary material for: Sex-specific effects of peptidyl arginine deiminase 4 deficiency in the cafeteria diet-induced obesity-associated metabolic complications
Source: Front Endocrinol (Lausanne). 2026 Jan 2;16:1694559. doi: 10.3389/fendo.2025.1694559 (PMC12807990; doi:10.3389/fendo.2025.1694559)
Supplement: Supplementary file 3 [file DataSheet3.docx]

Supplementary Material

Table 1: **The composition of the cafeteria diet.**

|  | **Food item** | **Type** | **Texture** | **Energy (kcal/100g)** | **Protein (g/100g)** | **Sugar (g/100g)** | **Fat (g/100g)** | **Saturated fat (g/100g)** | **Fiber (g/100g)** |
| --- | --- | --- | --- | --- | --- | --- | --- | --- | --- |
| **Menu 1** | Salty sticks | Salty | Crunchy | 413 | 10 | 76 | 7 | 0,9 | N/A |
|  | Caramel snack | Sweet | Rigid | 7.5 | 4.1 | 65 | 24 | 14 | 2 |
|  | Chocolate donut | Sweet | sweet | 454 | 5,2 | 48 | 26 | 11 | 2,2 |
|  | Gold bears | Sweet | Chewy | 30.11 | 7 | 75 | 0,1 | 0,1 | N/A |
|  | Salami | Ultra-processed | Soft | 597 | 25 | 2,8 | 54 | 19,3 | N/A |
| **Menu 2** | Cheese crackers | Salty | Crunchy | 507 | 8.1 | 5,1 | 23 | 2,1 | 2,3 |
|  | Chocolate-vanilla biscuits | Sweet | Crunchy | 475 | 5.1 | 68 | 19 | 5 | 3 |
|  | Chocolate cream | Sweet | Smooth | 538 | 6.1 | 58 | 31 | 11 | N/A |
|  | Marshmallow | Sweet | Soft | 336 | 5 | 77 | 0,5 | 0,1 | 0,7 |
|  | Cheese | Ultra-processed | Soft | 347 | 26 | 0,5 | 17 | 11 | N/A |


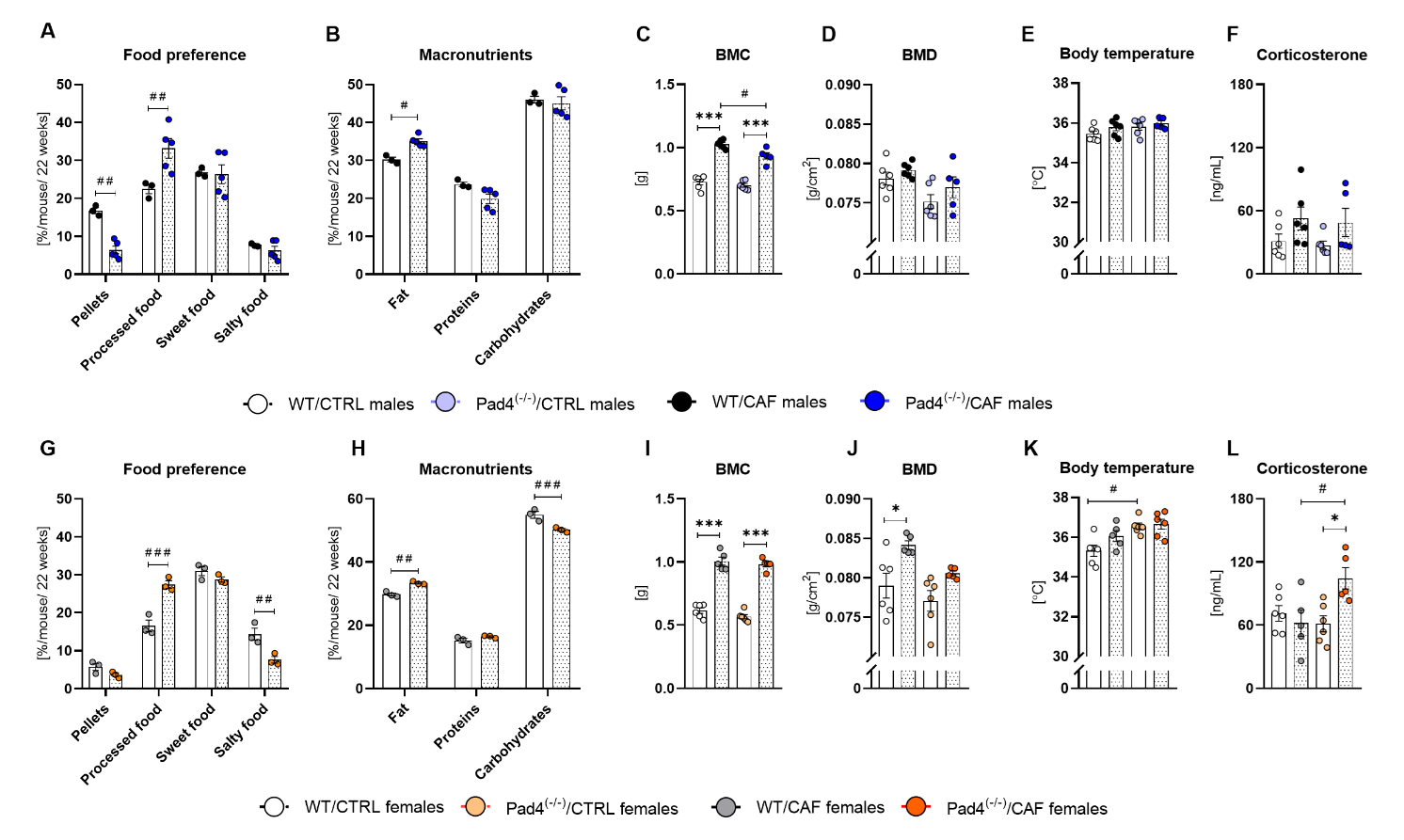


**Supplementary Figure 1. (A)** Food preference in males fed with CAF diet; **(B)** Proportion of macronutrient consumption in males fed with CAF diet; **(C)** Bone mineral content (BMC) in males; (**D**) Bone mineral density in males (BMD); **(E)** Body temperature in males; **(F)** Plasma corticosterone concentration in males; **(G)** Food preference in females fed with CAF diet; (**H**) Proportion of macronutrient consumption in females fed with CAF diet; **(I)** Bone mineral content (BMC) in females; Bone mineral density in females (BMD); **(K)** Body temperature in females; **(L)** Plasma corticosterone concentration in females. WT/CTRL males: n=6; WT/CAF males: n=6; Pad4^(-/-)^/CTRL males: n=6; Pad4^(-/-)^/CAF males: n=5; WT/CTRL females: n=6; WT/CAF females: n=5; Pad4^(-/-)^/CTRL females: n=6; Pad4^(-/-)^/CAF females: n=5. Statistical analysis: 2-way ANOVA with the Bonferroni post-hoc test. Data are presented as mean ± SEM. **^*^**CAF vs. CTRL (for both genotypes); **^#^**WT vs. Pad4^(-/-)^ (for both diet groups); **^*/#^**p<0.05; ##p<0.01 **^***/###^**p<0.001.

**Supplementary Figure 2.** **Inflammatory cytokine concentrations in plasma**. **(A)** Inflammatory cytokines in males. **(B)** Inflammatory cytokines in females. IL-1α- interleukin 1-alpha; IL-1β- interleukin 1-beta; TNF-α- tumor necrosis factor alpha; IL-6- interleukin 6; IL-10- interleukin; MCP-1- monocyte chemoattractant protein-1; IL-17A- interleukin 17A; IL-23- interleukin 23; interleukin 27 (IL-27) concentrations in males; INF-β- interferon beta; INF-γ- interferon gamma GM-CSF- granulocyte-macrophage colony-stimulating factor; IL-12p70- interleukin 12p70. WT/CTRL males: n=6; WT/CAF males: n=6; Pad4^(-/-)^/CTRL males: n=6; Pad4^(-/-)^/CAF males: n=5; WT/CTRL females: n=6; WT/CAF females: n=5; Pad4^(-/-)^/CTRL females: n=6; Pad4^(-/-)^/CAF females: n=5. Statistical analysis: Multiple T-test. Data are presented as decadic logarithms of cytokine concentrations.
